# Life in the cystic fibrosis upper respiratory tract influences competitive ability of the opportunistic pathogen *Pseudomonas aeruginosa*

**DOI:** 10.1098/rsos.180623

**Published:** 2018-09-19

**Authors:** Jeffrey J. Bara, Zachary Matson, Susanna K. Remold

**Affiliations:** 1 Department of Biology, University of Louisville, Louisville, KY, USA; 2 Department of Biology, Shenandoah University, Winchester, VA, USA

**Keywords:** cystic fibrosis, bacteriocins, *Pseudomonas aeruginosa*, host adaptation

## Abstract

Understanding characteristic differences between host-associated and free-living opportunistic pathogens can provide insight into the fundamental requirements for success after dispersal to the host environment, and more generally into the ecological and evolutionary processes by which populations respond to simultaneous selection on complex interacting traits. We examined how cystic fibrosis (CF)-associated and environmental isolates of the opportunistic pathogen *Pseudomonas aeruginosa* differ in the production of an ecologically important class of proteinaceous toxins known as bacteriocins, and how overall competitive ability depends on the production of and resistance to these bacteriocins. We determined bacteriocin gene content in a diverse collection of environmental and CF isolates and measured bacteriocin-mediated inhibition, resistance and the outcome of competition in a shared environment between all possible pairs of these isolates at 25°C and 37°C. Although CF isolates encoded significantly more bacteriocin genes, our phenotypic assays suggest that they have diminished bacteriocin-mediated killing and resistance capabilities relative to environmental isolates, regardless of incubation temperature. Notably, however, although bacteriocin killing and resistance profiles significantly predicted head-to-head competitive outcomes, CF and environmental isolates did not differ significantly in their competitive ability. This suggests that the contribution of bacteriocins to competitive ability involves selection on other traits that may be pleiotropically linked to interference competition mediated by bacteriocins.

## 1. Introduction

Many significant human pathogens are opportunistic: they are free-living organisms found throughout the environment that are only able to successfully infect hosts that are immunocompromised or have a condition that predisposes them to infection [1]. For invading environmental organisms, the host can be an alien, hostile environment that presents a diverse range of challenges (e.g. antibiotic exposure, host immune response, temperature and pH), which must be successfully overcome for the invading organism to persist and survive in the host [2,3]. Following host colonization, such opportunistic pathogens routinely undergo extensive adaptations in response to the abiotic and biotic factors they experience in the host environment [4–7].

Understanding characteristic phenotypic differences between host-associated and environmental isolates of an opportunistic pathogen can provide insight into the fundamental requirements for success after dispersal to the host environment and the ecological and evolutionary processes by which populations respond to simultaneous selection on complex interacting traits. In this study, we investigate phenotypic differences between isolates of the opportunistic pathogen *Pseudomonas aeruginosa* collected from individuals with cystic fibrosis (CF) and environmental isolates that are not associated with this pathogenic lifestyle.

CF is a debilitating genetically inherited disease that results in defective chloride ion transport across epithelial cell surfaces [8–10]. Individuals with CF are highly susceptible to chronic polymicrobial respiratory infections due to the production of sticky, dehydrated mucus lining the lung, which reduces ciliary function and the effectiveness of the innate immune system [2,3,10]. *Pseudomonas aeruginosa* is the most common bacterial infection in individuals with CF: approximately 80% of individuals are chronically infected by age 20 [10]. While person-to-person transmission of *P. aeruginosa* can occur [11,12], the majority of infections are probably caused by isolates acquired from the environment [13–15].

Within the CF upper respiratory tract, *P. aeruginosa* is exposed to selective pressures that drive genetic and phenotypic diversity within infecting isolates over time [2,3,10]. Longitudinal sampling of CF sputum from chronically infected individuals indicates that independent lineages of *P. aeruginosa* often display parallel evolution [16], accumulating mutations that affect a similar set of traits including mucoid colony morphology, antibiotic resistance, lipopolysaccharide (LPS) structure, quorum sensing and the type III secretion system [17]. Friman *et al*. [18] found that relative to intermittent CF isolates, chronic CF isolates were less resistant to bacteriophage infection and had a reduced ability to kill a ciliate predator, suggesting that adaptation to the CF upper respiratory tract may be associated with a trade-off for survival in environmental habitats. However, the effect of life in the CF lung on the competitive ability of *P. aeruginosa* within and outside of the host remains poorly understood.

Competitive interactions among bacteria, including *P. aeruginosa,* are significantly influenced by the production of bacteriocins [19,20]. These antimicrobial toxins are known as pyocins when produced by *P. aeruginosa* [21]*.* Previous studies have found that CF isolates have a distinct pyocin phenotype relative to clinical non-CF isolates [22] and that over time chronic CF isolates often lose their ability to produce pyocin [23]. However, support has been found for both higher [24] and lower [25,26] rates of pyocin-mediated inhibition by clinical isolates relative to environmental isolates, leaving the effect of host association on pyocin production unclear.

It is important to note that pyocin production is one trait contributing to the outcome of interference competition, but is only one of many possible contributors to the ultimate outcome of competitive interactions. Additional factors may influence interference competition, such as contact-dependent or secretion system-mediated killing*,* and traits associated with resource use competition may also play a role. Therefore, it is important to consider the relationship between pyocin phenotype and competition affected by the full suite of traits at play in a common environment (herein called ‘overall competition’), in evaluating pyocins' ultimate effect on fitness.

In this study, we test four independent but related predictions regarding how life in the CF upper respiratory tract affects the pyocin phenotype and competitive ability of *P. aeruginosa*: (i) CF isolates are more susceptible to pyocin-mediated inhibition and inhibit the growth of other *P. aeruginosa* isolates at a lower rate relative to environmental isolates, (ii) the number of pyocin genes encoded by CF and environmental *P. aeruginosa* isolates explains their pyocin-mediated phenotypes, (iii) the ability to inhibit other *P. aeruginosa* isolates significantly affects the outcome of competitive interactions, and (iv) environmental isolates are competitively superior relative to CF isolates. Additionally, because the outcome of assays that assess pyocin inhibition and overall competitive outcomes could be temperature-dependent, we performed our experiments at the CF isolates’ ‘home environment’ temperature, 37°C (the temperature in the human lung), and at 25°C. Although environmental isolates experience a range of temperatures, 25**°**C is within the ‘home environment’ range experienced by most household environmental isolates, and outside that of CF isolates.

*A priori*, it could be expected that the outcome of interactions at the 25°C and 37°C might differ because these isolates are unlikely to experience both temperatures on a regular basis, and the outcome of evolution in homogeneous environments is often specialization [27,28]. Such specialization might result in each type of isolate performing best at its native temperature. However, because previous studies have found that environmental isolates were able to inhibit CF isolates at 37**°**C, perhaps because adaptation to the CF lung resulted in a significant reduction of pyocin production, it is also possible that environmental isolates can outperform CF isolates regardless of temperature.

## 2. Material and methods

### 2.1. *Pseudomonas aeruginosa* isolates

This study included 116 *P. aeruginosa* isolates (electronic supplementary material, table S1). The 86 environmental *P. aeruginosa* isolates used in this study were collected from sites in and around 15 homes in the Louisville, KY metropolitan area (detailed collection and species designation methods are described in [29,30]). Three of these 86 environmental isolates were collected from the upper respiratory tract of healthy individuals that did not have CF [29], and the rest are predominantly from household drains. Among the 30 CF isolates, 11 were obtained during the same multi-year sampling effort [30], the other 19 CF isolates are from de-identified CF sputum samples obtained from Norton's Hospital located in Louisville, KY (kindly provided by Dr Alan Junkins; electronic supplementary material, table S1). Because a frequent and comprehensive sampling regime is necessary to ascertain the precise length of time that particular genotype has spent in the lung [31], the infection duration of each CF isolate in our collection is unknown. *Pseudomonas aeruginosa* type-isolate PAO1 and the PAO1 *prtR^S162A^* mutant, which has an inactive transcriptional regulator that results in the bacterium being unable to induce pyocin expression [32], were used as positive and negative controls, respectively, in the pyocin inhibition and cross-streak assays. The PAO1 *prtR^S162A^* mutant was kindly provided by Jon Penterman (MIT).

### 2.2. Materials

For all the experiments, we used lysogeny broth (LB) media and agar to culture bacteria (10 g tryptone, 5 g yeast extract and 5 g sodium chloride per litre of reverse osmosis water for the broth and an additional 10 g agar powder for LB agar).

### 2.3. Experimental protocols

#### 2.3.1. Pyocin inhibition assay

We used an all-by-all agar spot assay to determine the ability of a *P. aeruginosa* isolate to inhibit the growth of another *P. aeruginosa* isolate through the production of pyocin at 25°C and 37**°**C [25]. At each temperature, all possible pairwise interactions among a set of 29 isolates (bolded isolates; electronic supplementary material, table S1) were tested simultaneously, and the entire assay was replicated six times at 37°C and five times at 25°C. Overnight cultures of each isolate were grown in 2 ml of LB broth with shaking at 250 r.p.m. at either 25°C or 37°C. Stationary phase cultures were subsequently split into two separate 2 ml deep 96-well plates, 10 μl of bacterial culture was added to 1 ml fresh LB broth, and the plates were incubated overnight with shaking at 250 r.p.m. Mitomycin-C (MMC, Fisher), a DNA damaging agent known to induce pyocin production [25,33], was added at a final concentration of 0.01 µg ml^−1^ to one of the 96-well plates to induce pyocin production. After 24–30 h, 2 µl of stationary phase culture from each isolate exposed to MMC (producer isolate) was spotted in triplicate onto LB agar plates and then incubated at 25°C or 37°C for 6–7 h. Chloroform (800 µl) was added to the surface of each LB agar plate to kill the bacterial colonies, and each plate was allowed to air dry for at least 30 min. A 5 ml volume of LB top agar inoculated with 100 µl of the bacterial culture that was not exposed to MMC (indicator isolate) was gently vortexed and poured evenly over the agar surface plate to create a bacterial lawn. Following an overnight incubation at 25°C or 37°C, each plate was assessed for the presence of visible zones of growth inhibition (electronic supplementary material, figure S1).

#### 2.3.2. Pyocin haplotyping

The pyocins produced by *P. aeruginosa*, are divided into three groups according to their structure and function. There are seven characterized S-type pyocins (S1–S6, AP41) that can be distinguished by their active domain function: DNase (S1–S3, S6, AP41), tRNase (S4), pore-forming activity (S5) and their receptor-mediated domains [34]. Each S-type pyocin has an associated immunity gene, though S1 and S2 share an immune gene. R- and F-type pyocins are derived from ancestral bacteriophage tails and kill target cells through membrane depolarization [33,34]. R- and F-type pyocins do not have associated immunity genes; rather resistance or susceptibility is primarily determined by LPS structure [33–35]. Using a PCR-based approach, we screened for the presence of genes in the R-type (PRF 10) and F-type (PRF 31 and PRF 38) pyocin cluster [33], as well as seven characterized S-type pyocins and their associated immunity genes. We did not screen for the pyocin S5 immune gene, for which we were unable to find a published primer set [36–39]. Primer sequences and amplicon sizes for each pyocin gene are provided in electronic supplementary material, table S2.

Genomic DNA was extracted from each *P. aeruginosa* isolate according to the protocol described by Spilker *et al*. [40]. PCR reactions were carried out in a 15 μl volume with a final concentration of 25 U ml^−1^ Taq polymerase (New England Biolabs), 1× Taq buffer with 1.5 mM MgCl_2_, 0.2 mM dNTPs (Fisher Scientific), 0.5 mM MgCl_2_ and 0.2 µM forward and reverse primers, and PCR products were visualized on a 2% agarose gel containing ethidium bromide. For each isolate, we independently confirmed the PCR results for each pyocin gene using a separate DNA extraction. If there was ambiguity between the results of the first two extractions a PCR reaction was carried out using a third DNA extraction to resolve the disagreement. If we found that an isolate encoded either the PRF 31 or PRF 38, it was concluded that the isolate encoded the F-type pyocin gene [33].

#### 2.3.3. Cross-streak assay

We used an all-by-all cross-streak assay to assess the overall competitive ability of the 29 isolates when grown in the absence of MMC. This assay is designed to include all components of both interference and resource use competition at play in a common garden agar plate. All possible pairwise interactions were tested with threefold replication. Overnight cultures of each isolate were grown in 2 ml of LB broth with shaking at 250 r.p.m. at either 25°C or 37°C. Cell density was measured at OD600, and all cultures were standardized to an OD of 0.3. On LB agar plates, one *P. aeruginosa* isolate was streaked three times vertically on an LB agar plate (focal isolate) and allowed to air-dry for 5–10 min. The competitor isolates were then streaked at a 90° angle across each vertical streak using a new applicator each time. Plates were then incubated at either 25°C or 37°C for 24–36 h, and the outcome of the interaction was determined by observation of the growth pattern at the intersection of the two isolates (electronic supplementary material, figure S2). Cross-streaks involving PAO1 and *prtR^S162A^* were performed to directly assess how the absence of pyocin production influences competitive ability.

### 2.4. Data analysis

#### 2.4.1. Effect of life in the CF lung on rates of pyocin-mediated inhibition and cross-streak outcomes

These models and those of results of the cross-streak assay described below (§2.4.3) were performed in PROC GLIMMIX SAS v. 9.4. [41]. Analogous generalized linear mixed models with the Laplace approximation for the marginal likelihood were used to model growth inhibition or cross-streak outcome as a binomially distributed response variable. The probability of either growth inhibition by the producer isolate (pyocin-mediated inhibition assay) or of winning a cross-streak trial by the focal isolate (cross-streak competition assay) was determined using a logit link function, and all pairwise comparisons were performed with Tukey's correction for multiple comparisons. We fitted nested random factors with and without the assumption of equal variances among levels of the grouping variable to test for significant differences in the variance between the two grouping variable categories using likelihood ratio tests generated from sub-models in which the parameter of interest was removed from the model. Analyses were performed separately for experiments conducted at 25°C and 37°C.

In the pyocin-mediated inhibition models, we assessed the effects of the fixed variables producer and indicator source of isolation (environment or CF) and their interaction. Producer and indicator isolate nested within source and their interaction were included as random effects. In a parallel model of the outcome of the cross-streak assay, we assessed the effects of focal and competitor sources of isolation (environment or CF) and their interactions included as fixed factors and producer and indicator isolate as random effects. The interaction between producer and indicator isolates was not included due to failure of model convergence.

We used a Fisher's exact test to assess differences in pyocin-mediated inhibition and susceptibility between PAO1 and the PAO1 *prtR^S162A^* mutant. We combined the PAO1 and *prtR*^S162A^ data from 25°C and 37°C for all statistical analyses involving these two isolates.

#### 2.4.2. Pyocin haplotype

We used a non-parametric Kruskal–Wallis test (PROC NPAR1WAY) to assess the differences in the total number of pyocin genes, the number of toxin genes and the number of immunity genes encoded by environmental and CF *P. aeruginosa* isolates. We corrected for multiple comparisons using the Bonferroni adjustment. To determine whether specific pyocin genes were encoded by environmental or CF isolates at a significantly higher or lower frequency than expected to occur randomly, we used Fisher's exact tests and corrected for multiple comparisons using the Bonferroni adjustment.

#### 2.4.3. Relationship between pyocin inhibition and cross-streak assay outcomes

We investigated the ability of the pyocin inhibition assay to predict the outcome of the cross-streak assay. For each pair of interacting isolates, we determined whether the focal isolate had been able to inhibit the growth of that particular competitor isolate in at least three replicates. If so, that focal-competitor pairing was coded as a case of inhibition by that focal isolate, and otherwise was coded as non-inhibiting. We analogously assessed the ability of the competitor isolate to inhibit the focal isolate. We assessed the effects of the fixed variables focal isolate ability to inhibit competitor isolate, competitor isolate ability to inhibit the focal isolate and their interaction. Focal and competitor isolate nested within inhibition outcome were included as random effects. We used a Fisher's exact test to assess differences in the outcome of the cross-streak assay between PAO1 and the PAO1 *prtR^S162A^* mutant.

## 3. Results

### 3.1. Pyocin inhibition among environmental and CF isolates

As expected, PAO1 had a significantly higher (*p* < 0.001) inhibition rate relative to the *prtR^S162A^* mutant (electronic supplementary material, figure S3a). Based on rare growth inhibition by *prtR^S162A^* we estimate that the false positive rate of our inhibition assay was approximately 2%. The *prtR^S162A^* isolate was also significantly more susceptible to pyocin-mediated inhibition relative to PAO1 (*p* < 0.001) (electronic supplementary material, figure S3b). Overall, the observed difference in the inhibition and susceptibility rate between PAO1 and *prtR^S162A^* strongly suggests that pyocin and no other inhibitory compounds were responsible for the zones of inhibition that we observed in this assay.

**Figure 1.**
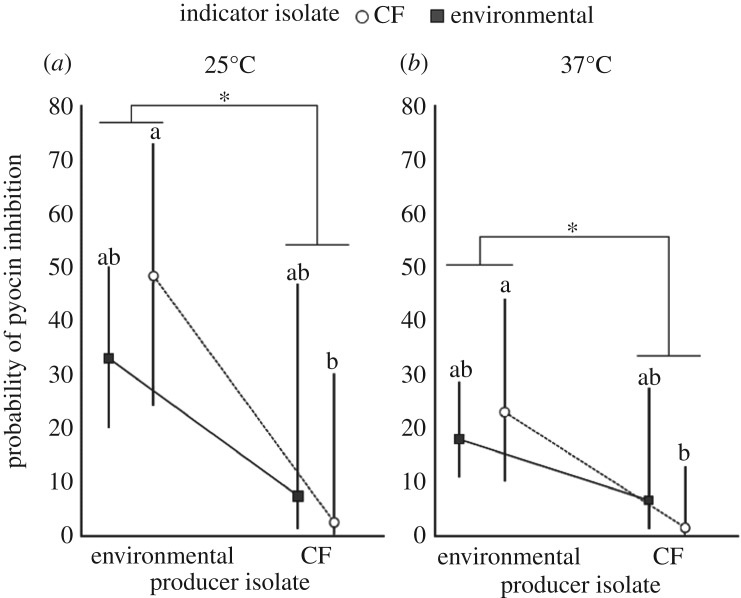
Probability of pyocin inhibition by source of isolation. Least-squares means with 95% confidence intervals of predicted inhibition as a function of producer and indicator source of isolation (environmental or CF) at (*a*) 25°C and (*b*) 37°C. Pairwise tests were corrected for multiple comparisons using the Tukey–Kramer adjustment. Different letters indicate significant differences in means.^n.s.^*p* > 0.1; ^∗^0.01 < *p* < 0.05.

We conducted bidirectional pyocin inhibition assays at both 25°C and 37°C to assess whether these results were temperature-dependent. Inhibition patterns were qualitatively consistent across temperatures (25°C and 37°C, chosen to reflect temperatures frequently experienced by environmental and CF isolates respectively), indicating no specialization on home temperatures, but rather overall superior inhibition by environmental isolates. Overall, environmental isolates inhibited other isolates significantly more frequently than CF isolates (Producer source main effect, 25°C: *p* = 0.04, 37°C: *p* = 0.03; electronic supplementary material, table S3; figure 1), but there was also significant variability among outcomes attributable to particular producer and indicator isolate pairings (Producer isolate (Producer source) × Indicator isolate (Indicator source), *p <* 0.001). Producer isolates also differed overall in their ability to inhibit (Producer isolate (Producer source) mean and variance effects; *p <* 0.001, electronic supplementary material, table S3; figure 1).

Despite significant variation among indicator isolates (Indicator isolate (Indicator source), electronic supplementary material, table S3), there was no overall difference in the susceptibility to pyocin inhibition between environmental and CF isolates (Indicator source main effect, 25°C: *p* = 0.67, 37°C: *p* = 0.34; electronic supplementary material table S3). This result was driven by the fact that environmental isolates do not differ in resistance to environmental versus CF isolates. However, after adjusting for multiple comparisons, we found that CF isolates were inhibited by environmental isolates significantly more often than by other CF isolates (Producer source × Indicator source, 25°C: *p* = 0.059, 37°C: *p* = 0.037; electronic supplementary material, table S3). Thus, our data suggest that CF isolates are both overall weaker pyocin producers and are less able to withstand inhibition by environmental isolates.

### 3.2. Pyocin haplotypes of environmental and CF isolates

**Figure 2.**
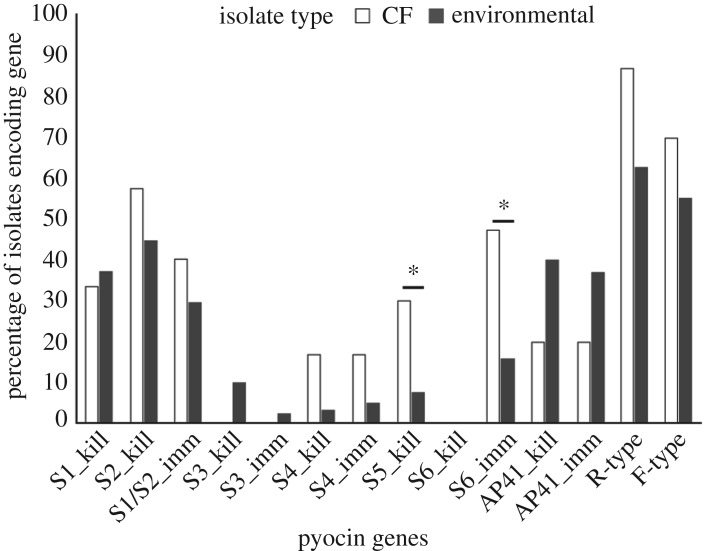
Frequency of pyocin genes found in isolates obtained from environmental and CF sources. ^n.s.^*p* > 0.1; ^∗^0.01 < *p* < 0.05.

**Figure 3.**
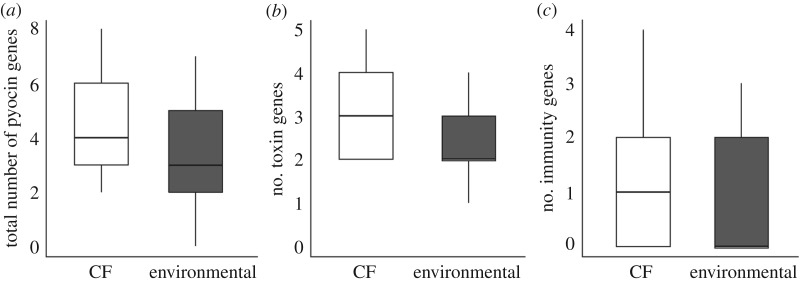
Average number of pyocin genes. (*a*) Total number of pyocin genes, (*b*) average number of toxin genes and (*c*) average number of immunity genes encoded by environmental and CF isolates.^n.s.^*p* > 0.1; ^∗^0.01 < *p* < 0.05.

We identified at least one pyocin gene in 115 of the 116 *P. aeruginosa* isolates included in this study (electronic supplementary material, table S6). After adjusting for multiple comparisons, the frequencies of the S6 immunity gene (*p* = 0.001) and the S5 toxin gene (*p* = 0.0027) were significantly higher in CF isolates relative to environmental isolates (figure 2). Notably, contrary to what might be expected based on their reduced pyocin killing and resistance capabilities, overall CF isolates encoded significantly more pyocin genes than environmental isolates (*H* = 6.054, *p* = 0.014; figure 3*a*). This result was primarily driven by the greater number of toxin genes encoded by CF isolates (*H* = 5.4216, *p* = 0.019; figure 3*b*), as there was no significant difference in the number of S-type immune genes encoded by CF and environmental isolates (*H* = 2.41, *p* = 0.12; figure 3*c*).

### 3.3. Pyocin haplotype diversity

Overall, the R- and F-type pyocins were the most commonly encoded genes, with a frequency of 67% and 58%, respectively. In comparison, S-type pyocin genes were encoded at a relatively lower frequency: no S-type pyocin was encoded by more than 50% of our isolates, and several genes (S3, S4 and S5) were found in less than 20% of our isolates. We identified 41 unique combinations of pyocin genes among our 116 isolates (electronic supplementary material, table S6), indicating that environmental and CF *P. aeruginosa* isolates will frequently possess a distinct arsenal of cytotoxic and immunity genes.

Another notable result was the frequent occurrence of ‘orphan genes’, which occur when the gene encoding the S-type cytotoxic or immunity protein is present, and the corresponding cytotoxic or immunity gene is absent. Approximately 27% (31/114) of the S-type immune genes and 45% (70/154) of the S-type cytotoxic gene encoded by isolates in this study were orphans. The S6 immune gene accounted for 90% of these instances of orphan immunity as it was found exclusively as an orphan immunity gene. The phenomenon of orphan cytotoxic genes was primarily driven by the number of orphan S1 and S2 pyocins, which accounted for approximately 85% of these instances.

### 3.4. Competition dependence on pyocin inhibition

**Figure 4.**
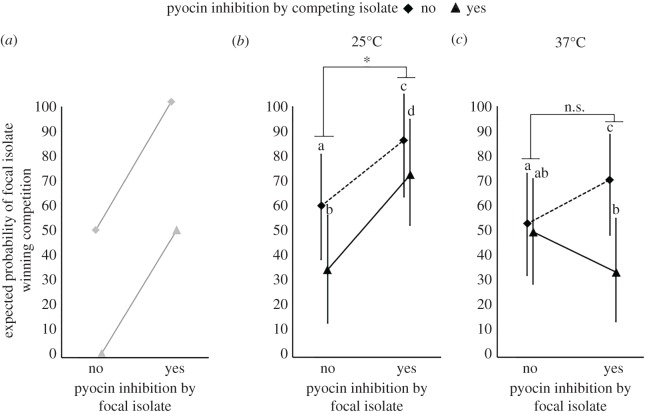
Probability that the focal isolate will outcompete the competitor with respect to inhibition phenotype. (*a*) Expected probability of a focal isolate winning a competition as a function of whether or not the focal and competitor isolates can inhibit the other. Least-squares means with 95% confidence intervals of predicted competition outcome as a function of whether or not the focal and competitor isolates can inhibit the other at (*b*) 25°C and (*c*) 37°C. Tests of pairwise comparisons were corrected for multiple comparisons using the Tukey–Kramer adjustment. Different letters indicate significant differences in means.^n.s.^*p* > 0.1; ^∗^0.01 < *p* < 0.05; ^∗∗^0.001 < *p* < 0.01; ^∗∗∗^*p* < 0.001.

The interpretation of the commonly used pyocin inhibition assay hinges on the extent to which pyocin production and susceptibility phenotypes accurately predict the outcome of overall competitive interactions that include the other traits contributing to interference and resource use competition in a shared environment. The fact that pyocin production contributes to competitive outcomes as assessed by our cross-streak assay was confirmed for our system using wild-type PAO1 and its *prtR*^S162A^ mutant. Overall, the *prtR*^S162A^ mutant was a significantly inferior competitor relative to PAO1 when streaked against the identical set of 29 *P. aeruginosa* isolates (*χ*^2^ = 175.34, *p* < 0.0001; electronic supplementary material, figure S4. Based on this result, the expected outcome of competition among isolates if pyocin inhibition and susceptibility were a strong predictor is shown in figure 4*a*.

Bidirectional cross-streak assays in the absence of MMC with the identical set of 29 isolates used in the pyocin inhibition assay at both 25°C and 37°C (electronic supplementary material, table S1) showed that the relationship between inhibition profile and competitive outcomes deviated from our expectation based on PAO1, and differed somewhat at the two temperatures. At 25°C, as expected, the focal isolate was significantly more likely to win the competition if it could inhibit the competitor (focal isolate inhibition main effect, *p* < 0.0001; electronic supplementary material, table S4; figure 4*b*). In addition, this was dependent on whether the competitor could inhibit the focal isolate: if the competing isolate could not inhibit the focal isolate, the focal isolate was more likely to outcompete it (competitor inhibition main effect, *p* < 0.0001; figure 4*b*).

In contrast to results at 25°C, where inhibition always increased a focal isolate's success in competition, at 37°C, this was true only when the competitor could not inhibit the focal isolate (focal isolate inhibition × competitor inhibition, *p* < 0.001; electronic supplementary material, table S4; figure 4*c*). In competitive interactions with competitors that could inhibit their growth, focal isolates' ability to outcompete was opposite to what was expected. Also, at both temperatures we found that even when the more successful isolate was the one predicted, the advantage observed was smaller than expected, further indicating that factors besides pyocin inhibition and resistance profiles affect the outcome of competition in this assay.

### 3.5. Competition among environmental and CF isolates

**Figure 5.**
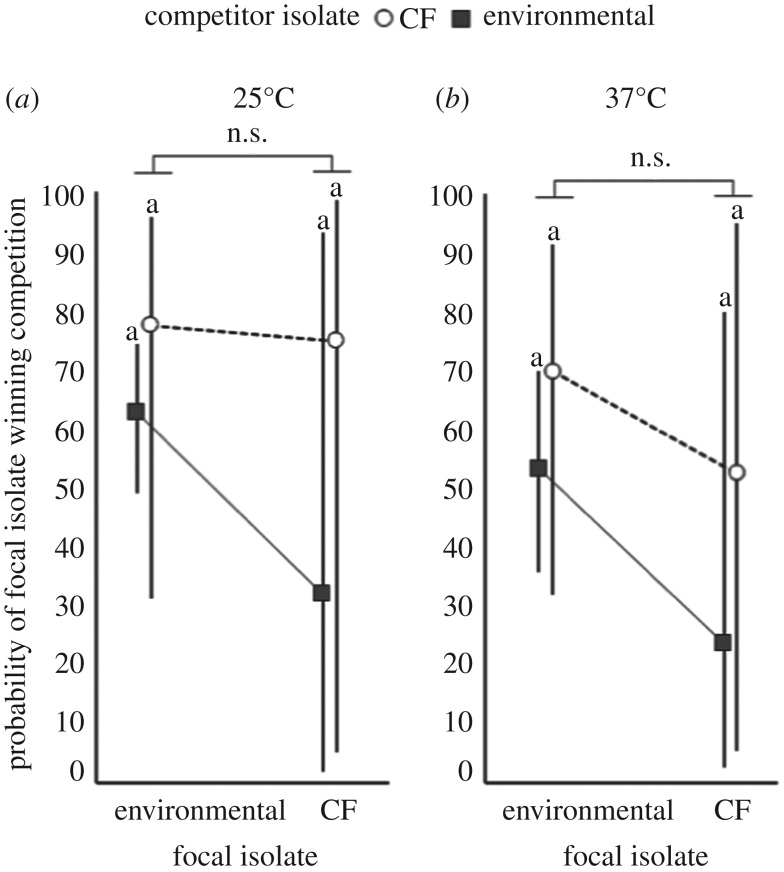
Probability that the focal isolate will outcompete the competitor with respect to source of isolate. Least-squares means with 95% confidence intervals of predicted competition outcome as a function of each player's source of isolation (environmental or CF) at (*a*) 25°C and (*b*) 37°C. There are no significant differences among means.

We constructed statistical models parallel to those used to analyse the pyocin inhibition assays and found that, unlike the outcome of the inhibition assays, the outcome of the cross-streak competition assay was poorly predicted by the source of isolation. Although there was a trend toward CF isolates exhibiting lower competitive ability and poorer ability to compete against environmental isolates, these differences were not statistically significant at either temperature (focal isolate source, competitor source and their interaction at both 25°C and 37°C; *p* > 0.1; electronic supplementary material, table S5; figure 5).

The difference in the pattern of inhibition probability and the probability of outcompeting another isolate is neither driven by higher experimental error rates nor by lack of power stemming from smaller predicted differences between inhibition and competition probabilities. In fact, the differences in competition success between CF and environmental isolates are greater than the differences in inhibition rates (figure 1). Rather, there is substantial variability among isolates and among pairings of isolates in both inhibition and competitive ability (significant mean and/or variance effects of focal isolate and competing isolate at both 25°C and 37°C; electronic supplementary material, table S5).

## 4. Discussion

How does life in the CF lung affect the pyocin phenotype and competitive ability of *P. aeruginosa*? We tested the following four independent but related hypotheses at two temperatures: (i) CF isolates are more susceptible to pyocin-mediated inhibition and inhibit the growth of other *P. aeruginosa* isolates through pyocin production at a lower rate relative to environmental isolates, (ii) the number of pyocin genes encoded by CF and environmental *P. aeruginosa* isolates explains their pyocin-mediated phenotypes, (iii) The ability to inhibit another *P. aeruginosa* isolate significantly affects the outcome of competitive interactions, and (iv) environmental isolates are competitively superior relative to CF isolates.

Our results supported our first and third hypotheses. We found a loss of bacteriocin-based killing and resistance in CF isolates, and a link between inhibition phenotype and competitive outcomes *in vitro,* though results of competition assays indicate that other factors besides inhibition phenotype also influence competitive outcomes. Our findings did not support our second and fourth hypothesis: the observed difference in pyocin phenotype between CF and environmental isolates was not attributable to the number of pyocin genes encoded by these isolates. Furthermore, the CF isolates we studied were not distinguishable from environmental isolates in their overall competitive ability, due in large part to their high variability concerning this trait. Coupled with the significant pyocin haplotype diversity found among both the environmental and CF isolates, this suggests that the link between capacity to inhibit through pyocins and overall competitive ability varies among isolates, and may involve selection on traits specifically relevant to the CF lung environment that are pleiotropically linked to bacteriocin killing and resistance and exert indirect selection on the latter.

### 4.1. Pyocin inhibition among environmental and CF isolates

CF isolates tended to be more susceptible to pyocin-mediated inhibition and inhibited the growth of other *P. aeruginosa* isolates at a lower rate than environmental isolates.

This result is in agreement with recent pyocin inhibition studies [25,26], and a longitudinal pyocin typing study [23]. Together, these studies suggest that a decrease in functional pyocin production and an increase in pyocin susceptibility is a common phenotypic change in CF isolates and could be a characteristic trait of host adaptation. These changes in pyocin inhibition and resistance phenotypes in CF isolates could occur directly, or indirectly due to selection acting on another trait, though these scenarios are not mutually exclusive.

Social dynamics [31,42,43] and *P. aeruginosa* population structure within the CF upper respiratory tract could directly affect the fitness benefits associated with contributions of pyocin-based inhibition to competitive ability. Direct selection against pyocin production may be a consequence of exposure to common stressors in the CF upper respiratory tract such as reactive oxygen species or the fluoroquinolone antibiotic ciprofloxacin as pyocin expression, because pyocin expression in response to these stressors may be associated with fitness costs [44–46].

Indirect selection on pyocin resistance could result from selection for reduced immunogenicity within the host. Pyocin susceptibility to R/F-type pyocin may be significantly altered in response to the loss of LPS B-band biosynthesis [35,44], which is commonly observed in CF isolates [17]. The observed difference in pyocin susceptibility between environmental and CF isolates could also be driven by differential expression or regulation of their pyocin genes. The role of gene expression on pyocin susceptibility is supported by our observation that the PAO1 *prtR^S162A^* mutant was 2.5-fold more susceptible to pyocin-mediated inhibition relative to PAO1. Given that the only difference between these two isolates is the inability of *prtR^S162A^* to express genes in the pyocin pathway, this result suggests that the expression of intracellular S-type immune proteins may have an important role in facilitating resistance to S-type pyocin-mediated killing. Given that our inhibition results were identical to previous pyocin studies [25,26,36,47] this result also provides additional evidence to suggest that pyocins were responsible for the growth inhibition that we observed in our study (electronic supplementary material, figure S1). However, our experimental design did not allow us to exclude bacteriophages as an alternative factor resulting in growth inhibition. Given that temperate phages are induced by exposure to mitomycin-C [33] and lytic activity by bacteriophage can significantly influence *P. aeruginosa* population dynamics [48] future studies should investigate the contribution of bacteriophage lysis to competitive dynamics.

We performed all phenotypic components of this study at both 37°C and at 25°C, because the shift from an environmental to a host-associated state is accompanied by a significant and abrupt change in environmental temperature among other abiotic factors, and temperature has been shown to affect *P. aeruginosa* gene expression [49]. We could not simultaneously conduct assays at different temperatures in the same incubators, but we note that overall patterns of inhibition differences were consistent between temperatures.

### 4.2. Pyocin haplotypes of environmental and CF isolates

We hypothesized that the number of pyocin genes encoded by CF and environmental *P. aeruginosa* isolates explains their pyocin-mediated phenotypes. However, we found that despite their lower inhibition rates and resistance to pyocins, CF isolates encoded a higher number of pyocin genes relative to environmental isolates. One explanation for this finding is that isolates that encode a higher number of pyocins could have an enhanced ability to colonize individuals with CF. The ability of *P. aeruginosa* to invade hosts is known to depend on the production of an array of secreted toxins, enzymes, redox cycling molecules, siderophores and exopolysaccharides [50]. Although pyocin production may be selected against over the course of a chronic infection, it could play an important role in early CF airway infections by freeing up niche space through the competitive removal of sensitive bacterial species colonizing the CF lung. S-type pyocin genes are known to be expressed early in CF airway infections [51], to display potent activity against biofilms [52], to be stable in murine models [53] and to be effective against some other bacterial species that may also be found in the CF lung, such as *Burkholderia cepacia* in addition to *P. aeruginosa* [36].

Our finding that CF isolates tended to encode more pyocin genes relative to environmental isolates is in disagreement with the only other comparable published study on this topic: Ghoul *et al*. [26] found that chronic and multiply chronic CF isolates encoded a significantly lower number of pyocin types relative to non-lung and acute CF isolates. There are several possible non-mutually exclusive explanations for this difference. First, Ghoul *et al*. [26] categorized their CF isolates by infection duration (acute, chronic and multiply chronic). We lacked the relevant metadata concerning our CF isolates to make this distinction, and it is possible that if we had been able to we would have found a similar trend. It is also possible that our collection of *P. aeruginosa* included few or no chronic CF isolates, or that characteristics of their environmental isolates and ours, which were obtained primarily from household drains, differ. Finally, their data analysis method differed from ours in that while they compared the number of distinct pyocin types between classes of isolates, we assessed differences in the number of pyocin genes. An analysis of the number of pyocin genes among classes of isolates in their supplementary data found no significant difference in this characteristic of haplotype diversity between environmental and CF isolates in their study.

It is surprising that we found so many cases in which S-type toxin genes were encoded without a corresponding immunity gene present and *vice versa,* because S-type pyocins are believed to be released as binary protein complexes with the smaller immune protein bound to the C-terminal of the large cytotoxic protein to prevent toxicity to the producer cell [21,34]. Nevertheless, we found 27% of the immune genes and 45% of the cytotoxic genes encoded by our isolates occurred as orphans (electronic supplementary material, table S6), and similarly, Ghoul *et al.* [26] also observed this phenomenon, finding that 14% of the immune genes and 35% of the S-type cytotoxic genes encoded by isolates in their study occurred as orphan genes. The incidence of orphan immunity genes is intuitive as such genes could confer a competitive advantage by allowing the bacterium to neutralize a broader range of toxins without imposing the biological cost of toxin production and lysis [37]. However, the frequent presence of cytotoxic genes without a corresponding immunity defies the current understanding regarding how S-type pyocins are regulated and expressed. Given that we never observed any self-inhibition, it is clear that there may be alternative mechanisms providing resistance to these cytotoxic proteins.

### 4.3. Overall competitive outcomes

Eliminating pyocin production in PAO1 significantly affected the outcome of competitive interactions as measured by our cross-streak competition assay in a common environment and allowing cell-to-cell contact. However, we found that among a diverse collection of environmental and clinical isolates, the outcome of competitive interactions was only partially predictable based on results of pairwise inhibition assays. In particular, where a clear competitive winner was expected, the likelihood of this success was always less than 100%, and the probability of a non-inhibiting focal isolate outcompeting an inhibiting competitor was much higher than expected. This suggests that other factors, such as differences in resource competition ability, contact-dependent killing or secretion system-mediated killing, may have influenced the outcome of these interactions. A non-mutually exclusive explanation is that the capacity to inhibit when induced by MMC does not entirely predict the pattern or strength of induction of pyocin production in the absence of MMC, when a competitor instead induces pyocin production. Such differences may lead to discrepancies between outcomes of the two assays, primarily because the cross-streaking technique provides a different environment for the focal isolate, streaked onto the plate first, then for the competitor isolate. Furthermore, we found no evidence that overall competitive ability depended on either adaptation to home temperature conditions or overall differences between environmental or CF isolates. Instead, in our assays we found no significant differences in the outcome of competition between environmental and CF isolates at either temperature, indicating that differences affecting other components of competitive ability between environmental and CF isolates are not specific to the habitats they occupy. It also suggests that caution must be taken in equating results of inhibition studies with likely competitive superiority.

## 5. Conclusion

In this investigation of differences between environmental and CF isolates of the opportunistic pathogen *P. aeruginosa* we found that, consistent with our prediction and with previous results [25,26], relative to environmental isolates, CF isolates have a reduced capacity to kill and an increased susceptibility to pyocin-mediated inhibition by environmental isolates. However, contrary to our prediction, we found that relative to environmental isolates, CF isolates encoded more pyocin genes, indicating that elimination of these genes in the CF lung is not a common strategy and is not the driver of reduced pyocin-mediated killing and defence. Moreover, although we found that overall, the likelihood that a pair of isolates engaging in pyocin-mediated inhibition significantly predicted the outcome of competitive interactions among those isolates when interacting in a shared environment, our data did not support the hypothesis that CF isolates were competitively inferior to environmental isolates when other mechanisms of competition beyond pyocin-mediated inhibition were at play. Our results, therefore, provide further evidence for the loss of pyocin-based killing and pyocin resistance in CF isolates, but suggest that the underlying mechanism for this pattern is complex and may involve selection on other traits specifically relevant to the CF lung environment that are linked to pyocin expression and resistance and exert indirect selection on these traits.

## Supplementary Material

Supplemental figures and tables

## Data accessibility

Our raw data and SAS code are deposited at Dryad Digital Repository: http://dx.doi.org/10.5061/dryad.0m801b4 [54].
