# Supplementary material for: Life in the cystic fibrosis upper respiratory tract influences competitive ability of the opportunistic pathogen Pseudomonas aeruginosa
Source: R Soc Open Sci. 2018 Sep 19;5(9):180623. doi: 10.1098/rsos.180623 (PMC6170537; doi:10.1098/rsos.180623)
Supplement: Supplemental figures and tables [file rsos180623supp1.docx]

**Life in the Cystic Fibrosis Upper Respiratory Tract Influences Competitive Ability of the Opportunistic Pathogen *Pseudomonas aeruginosa***

Jeffrey J. Bara^1*,^ Zachary Matson^1^, and Susanna K. Remold^1^

Supplemental Materials

Figures


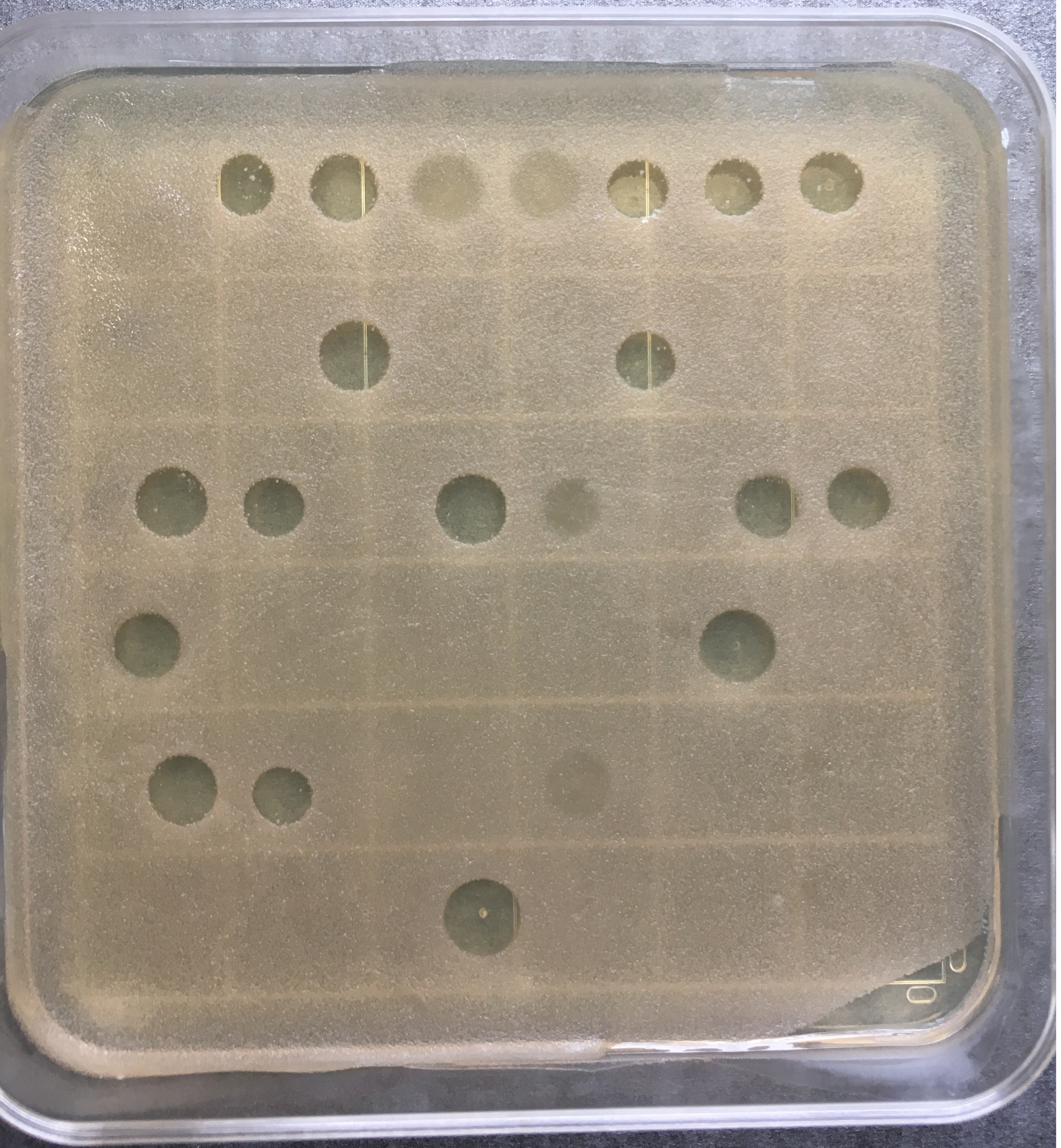


**Figure S1 pyocin inhibition plate.** Representative lawn of a *P. aeruginosa* indicator isolate growing on the surface of an LB agar plate that was spotted with *P. aeruginosa* producer cultures whose pyocin expression was induced through exposure to MMC. The formation of clear zones of inhibition is indicative of pyocin-mediated growth inhibition. The opaque or faint inhibition seen for some interactions is indicative of S-type pyocin mediated inhibition whereas the clear inhibition is indicative of R/F-type mediated inhibition.


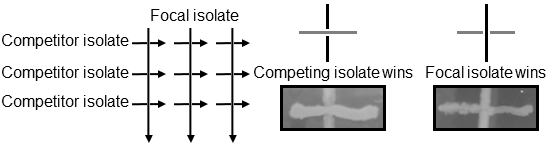


**Figure S2 Cross streak assay plate**. One *P. aeruginosa* isolate was streaked vertically onto LB agar plates followed by cross-streaking with different *P. aeruginosa* isolates. After an incubation period at 25 or 37 °C, the growth pattern of the two isolates was used to determine the outcome of the competitive interaction.


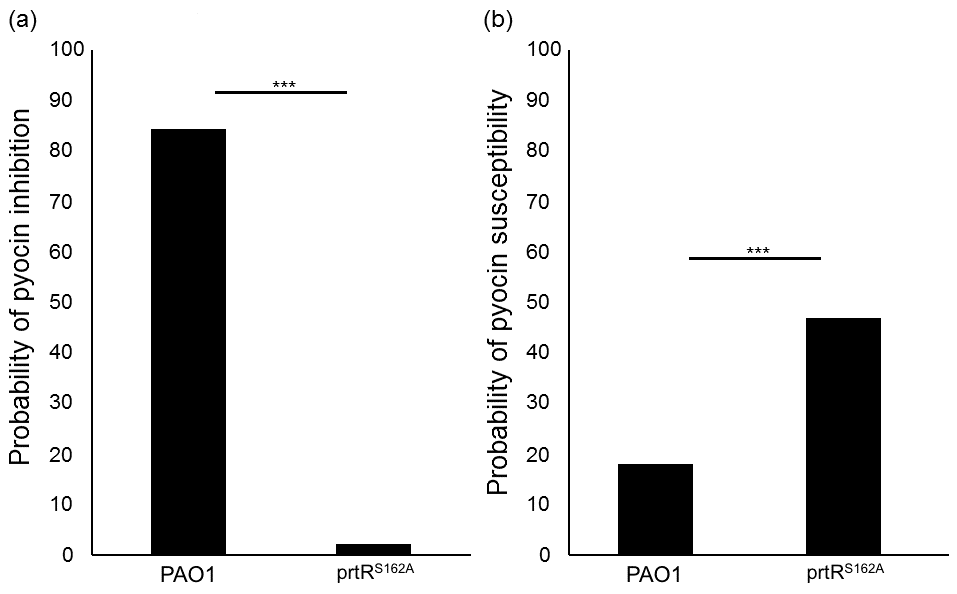


**Figure S3.** **Pyocin-mediated inhibition and pyocin resistance phenotypes of PAO1 and the *prtR*^S162A^ mutant**. (a) Probability that PAO1 or the *prtR*^S162A^ mutant inhibits isolates from a collection of 29 *P. aeruginosa* (bolded isolates, table S1). (b) Probability of being inhibited. ^NS^P>0.1; ^∗^0.01<P<0.05; ^∗∗^0.001<P<0.01; ^∗∗∗^P<0.001.


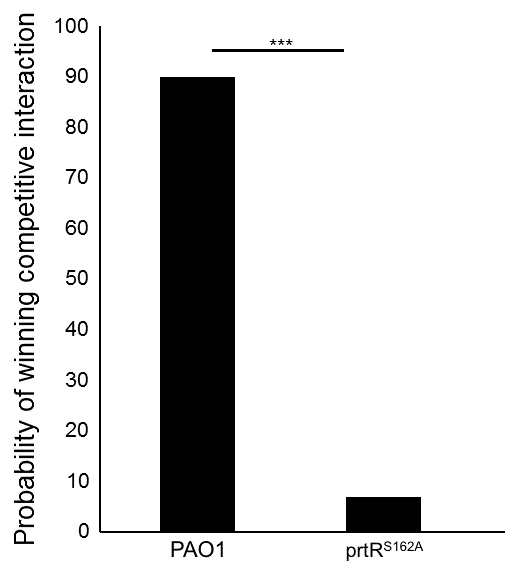


**Figure S4. Dependence of competition outcome on pyocin-production in PAO1 genetic background.** Probability that the PAO1 and the *prtR*^S162A^ mutant strain win a competition against isolates from a collection of 29 *P. aeruginosa* (bolded isolates, table S1). Results of competitions at 25 **°**C, and 37 **°**C are combined. ^NS^P>0.1; ^∗^0.01<P<0.05; ^∗∗^0.001<P<0.01; ^∗∗∗^P<0.001.

Tables

| **Isolate category** | **Isolate ID** | **Isolate source** |
| --- | --- | --- |
| CF-associated | 1055 | Throat |
| **CF-associated** | **1266** | **Mouth** |
| **CF-associated** | **1348** | **Sputum** |
| **CF-associated** | **1427** | **Throat** |
| CF-associated | 1790 | Mouth |
| CF-associated | 1794 | Sputum |
| CF-associated | 1814 | Sputum |
| CF-associated | 2092 | Sputum |
| **CF-associated** | **2552** | **Throat** |
| **CF-associated** | **3110** | **Sputum** |
| **CF-associated** | **3233** | **Throat** |
| CF-associated | 16-001 | Sputum |
| CF-associated | 16-002 | Sputum |
| CF-associated | 16-003 | Sputum |
| CF-associated | 16-004 | Sputum |
| CF-associated | 16-005 | Sputum |
| CF-associated | 16-006 | Sputum |
| CF-associated | 16-007 | Sputum |
| CF-associated | 16-008 | Sputum |
| CF-associated | 16-009 | Sputum |
| CF-associated | 16-096 | Sputum |
| CF-associated | 16-097 | Sputum |
| CF-associated | 16-098 | Sputum |
| CF-associated | 16-099 | Sputum |
| CF-associated | 16-100 | Sputum |
| CF-associated | 16-101 | Sputum |
| CF-associated | 16-102 | Sputum |
| CF-associated | 16-103 | Sputum |
| CF-associated | 16-104 | Sputum |
| CF-associated | 16-105 | Sputum |
| **Environmental** | **1005** | **Sputum (Non-CF)** |
| **Environmental** | **1027** | **Bathtub drain** |
| **Environmental** | **1062** | **Kitchen countertop** |
| Environmental | 1068 | Bathtub drain |
| **Environmental** | **1078** | **Kitchen sink brush** |
| **Environmental** | **1081** | **Kitchen sink brush** |
| Environmental | 1091 | Bathtub drain |
| Environmental | 1106 | Outdoor standing water |
| Environmental | 1150 | Bathroom sink drain |
| Environmental | 1162 | Bathroom sink drain |
| **Environmental** | **1169** | **Nose (Non-CF)** |
| **Environmental** | **1172** | **Kitchen sink brush** |
| **Environmental** | **1173** | **Kitchen garbage disposal** |
| Environmental | 1177 | Bathtub drain |
| **Environmental** | **1258** | **Kitchen sink drain** |
| **Environmental** | **1274** | **Kitchen sink drain** |
| Environmental | 1276 | Bath toy |
| Environmental | 1421 | Outdoor garden hose |
| Environmental | 1435 | Kitchen sink drain |
| Environmental | 1439 | Bath toy |
| Environmental | 1505 | Bathtub drain |
| **Environmental** | **1507** | **Bathtub drain** |
| **Environmental** | **1536** | **Bathroom sink drain** |
| Environmental | 1537 | Bathroom sink drain |
| Environmental | 1538 | Bathroom sink drain |
| Environmental | 1582 | Bathroom countertop |
| **Environmental** | **1607** | **Kitchen sink drain** |
| Environmental | 1609 | Bathroom sink drain |
| **Environmental** | **1618** | **Kitchen trash container** |
| Environmental | 1831 | Bathroom surface |
| **Environmental** | **1845** | **Bathroom sink drain** |
| **Environmental** | **1904** | **Throat (Non-CF)** |
| Environmental | 1943 | Outdoor bird bath |
| **Environmental** | **1952** | **Kitchen garbage disposal** |
| Environmental | 2004 | Outdoor trash can |
| Environmental | 2048 | Bath toy |
| Environmental | 2151 | Bathroom sink drain |
| Environmental | 2230 | Bathroom sink drain |
| Environmental | 2253 | Bathroom sink drain |
| Environmental | 2299 | Kitchen trash container |
| **Environmental** | **2304** | **Kitchen drying rack** |
| Environmental | 2361 | Outdoor bird bath |
| Environmental | 2415 | Bathroom sink drain |
| Environmental | 2500 | Kitchen garbage disposal |
| Environmental | 2521 | Bathroom sink drain |
| Environmental | 2544 | Bathtub drain |
| Environmental | 2562 | Bathtub drain |
| Environmental | 2563 | Bathroom sink drain |
| Environmental | 2578 | Bathtub drain |
| **Environmental** | **2588** | **Kitchen sink brush** |
| **Environmental** | **2590** | **Kitchen sink drain** |
| Environmental | 2594 | Bathtub drain |
| Environmental | 2597 | Bathroom countertop |
| Environmental | 2604 | Bathtub drain |
| Environmental | 2605 | Bathroom sink drain |
| Environmental | 2620 | Kitchen countertop |
| Environmental | 2629 | Bathtub drain |
| Environmental | 2630 | Bathtub drain |
| Environmental | 2633 | Bathroom sink drain |
| Environmental | 2670 | Bathroom showerhead |
| Environmental | 2674 | Bathroom sink drain |
| **Environmental** | **2684** | **Kitchen sink drain** |
| Environmental | 2696 | Bathtub drain |
| Environmental | 2704 | Bath toy |
| Environmental | 2785 | Bathroom sink drain |
| Environmental | 2836 | Bathroom sink drain |
| Environmental | 2877 | Bathroom sink drain |
| Environmental | 2967 | Bathroom sink drain |
| Environmental | 3061 | Bathroom sink drain |
| Environmental | 3091 | Child's spoon |
| Environmental | 3100 | Bathtub drain |
| Environmental | 3151 | Bathroom sink drain |
| Environmental | 3173 | Bathtub drain |
| Environmental | 3199 | Bathroom sink drain |
| **Environmental** | **3209** | **Bathroom sink drain** |
| **Environmental** | **3232** | **Bathroom sink drain** |
| Environmental | 16-012 | Bathroom sink drain |
| Environmental | 16-013 | Kitchen sink drain |
| Environmental | 16-019 | Bathroom sink drain |
| Environmental | 16-020 | Kitchen sink drain |
| Environmental | 16-023 | Bathroom sink drain |
| Environmental | 16-046 | Bathroom sink drain |
| Environmental | 16-054 | Bathroom sink drain |
| Environmental | 16-055 | Kitchen sink drain |
| Environmental | 16-064 | Bathroom sink drain |
| Environmental | 16-065 | Bathroom sink drain |

**Table S1. 116 P. aeruginosa isolates from Human and Environmental sources.** Pyocin haplotypes of all isolates were determined. Isolates in bold (n=29) were used in the pyocin inhibition and cross-streak assay.

| Primer set | Pyocin gene(s) | Primer pair | Primer sequence (5'-3') | Product size (bp) | Reference |
| --- | --- | --- | --- | --- | --- |
| 1 | S1 kill | Forward | ATGGCACGACCCATTGCTGACCTTA | 256 | [34] |
|  |  | Reverse | CATCTCGGAACTGAAGCTCGATCTC |  |  |
| 2 | S2 kill | Forward | ATGGCTGTCAATGATTACGAAC | 404 | [34] |
|  |  | Reverse | TGAGGAAAGTCTGAAGCCGT |  |  |
| 3 | S1/S2 immunity | Forward | ATGAAGTCCAAGATTTCCG | 264 | [34] |
|  |  | Reverse | CTAACCGGCCTTAAAGCCA |  |  |
| 4 | S3 kill | Forward | ATGGCTGATGCACCACCG | 854 | [34] |
|  |  | Reverse | TCGTTGACGATCTGCTTGAC |  |  |
| 5 | S3 immunity | Forward | ATGGAGAAGAAGCTGATCGTT | 462 | [34] |
|  |  | Reverse | CTATTTAGAACCAAGAAGAGC |  |  |
| 6 | S4 kill and immunity | Forward | GGAATTCCATATGACAAATAATAGTGCGCCACCAC | 2633 | [37] |
|  |  | Reverse | CCGCTCGAGTTATTTTCTGGAGGCAATTGTTAC |  |  |
| 7 | S4 immunity | Forward | AGGCAATGGGAAGATGTGG | 323 | [37] |
|  |  | Reverse | CCTCTGTACTCTCTTTCGC |  |  |
| 8 | S5 kill | Forward | GGAATTCCATATGTCCAATGACAACGAAGTACCTGG | 1496 | [36] |
|  |  | Reverse | CGGGATCCTTGAGCTTTAAATACTATTGGGC |  |  |
| 9 | S6 kill | Forward | GTCTCCAGATCCGCATGAAT | 884 | [35] |
|  |  | Reverse | CGGAGCAGGATGGTAACTGT |  |  |
| 10 | S6 immunity | Forward | CCTAGCATCGGGAAATGATG | 127 | [35] |
|  |  | Reverse | TACTCCAATCCAACCGGAAG |  |  |
| 11 | AP41 kill | Forward | ATGAGCGACGTTTTTGACCTT | 261 | [34] |
|  |  | Reverse | GGCAGTGTTCGTGGATTTTT |  |  |
| 12 | AP41 immunity | Forward | ATGGATATTAAAAATAACCTT | 273 | [34] |
|  |  | Reverse | TTAGCCAGCCTTGAAGCCA |  |  |
| 13 | PRF10 (R-type) | Forward | AGTTGATCGGCTTCTGGCCAGG | 307 | [32] |
|  |  | Reverse | TGCGTCCACTCGACCAGCCAG |  |  |
| 14 | PRF 31 (F-type) | Forward | AAGCCTGGACAGTTCGGCACTGA | 224 | [32] |
|  |  | Reverse | TCAGTAGTGCTTCGTTGAGCTTGG |  |  |
| 15 | PRF 38 (F-type) | Forward | AGGTTCGGTTTCCGTCACGCTG | 394 | [32] |
|  |  | Reverse | GTAACTCAAGGCGTTGGCCGG |  |  |

**Table S2. PCR primer sets used in this study.**

| **Source** | **df^1^** | **Test statistic^2^** |
| --- | --- | --- |
| 25 °C | | |
| Producer source | 1, 27 | 4.65* |
| Indicator source | 1, 27 | 0.17^NS^ |
| Producer source x Indicator source | 1, 754 | 4.02* |
| Producer isolate (Producer source) [*means*] | 1 | 324.79*** |
| Producer isolate (Producer source) [*variances*] | 1 | 8.52 ** |
| Indicator isolate (Indicator source) [*means*] | 1 | 963.15*** |
| Indicator isolate (Indicator source) [*variances*] | 1 | 0.12^NS^ |
| Producer isolate (Producer source) x Indicator isolate (Indicator source) [*means*] | 1 | 933.84*** |
| 37 °C | | |
| Producer source | 1, 27 | 5.24* |
| Indicator source | 1, 27 | 0.91^NS^ |
| Producer source x Indicator source | 1, 754 | 5.07* |
| Producer isolate (Producer source) [*means*] | 1 | 183.45*** |
| Producer isolate (Producer source) [*variances*] | 1 | 7.32 ** |
| Indicator isolate (Indicator source) [*means*] | 1 | 260.32*** |
| Indicator isolate (Indicator source) [*variances*] | 1 | 0.02^NS^ |
| Producer isolate (Producer source) *Indicator isolate (Indicator environment) [*means*] | 1 | 516.84*** |

| ^1^df indicates degrees of freedom. Denominator df for *F* test is estimated using the between-within approximation. Df for likelihood ratio (LR) tests are equal to the difference in the number of parameters in the full and reduced models. |
| --- |
| ^2^Fixed effects are tested with approximate *F* tests. Random effects are tested using likelihood ratio (LR) tests; the LR test statistic is −2× (maximum likelihood of the test’s full model − maximum likelihood of the restricted model, from which the variance component being tested has been removed), and is distributed approximately chi-squared. In the tests of variance effects, variances are constrained to be equal in the reduced model. ^NS^P>0.1; ^∗^0.01<P<0.05; ^∗∗^0.001<P<0.01; ^∗∗∗^P<0.001. |

**Table S3. General linear mixed models assessing dependence of outcome of pairwise inhibition assays among *P. aeruginosa* on isolation source.** Sources (environment or CF) and their interactions are included as fixed factors and producer and indicator isolate and their interactions as random factors. Separate models are presented for experiments conducted at 25 °C and 37 °C.

| **Source** | | **df^1^** | | **Test statistic^2^** | |  |
| --- | --- | --- | --- | --- | --- | --- |
| 25 °C | | | | | |  |
| Focal isolate inhibition | | | 1, 1714 | | 66.21*** | |
| Competitor isolate inhibition | | | 1, 1714 | | 26.70*** | |
| Focal isolate inhib. x competitor isolate inhib.. | | | 1, 1714 | | 0.60^NS^ | |
| Focal isolate [means] | | | 1 | | 487.64*** | |
| Competitor isolate [*means*] | | | 1 | | 177.96*** | |
| 37 °C | | | | | | |
| Focal isolate inhibition | | | 1, 1714 | | 0.04^NS^ | |
| Competitor isolate inhibition | | | 1, 1714 | | 22.79*** | |
| Focal isolate inhib. x competitor isolate inhib. | | | 1, 1714 | | 17.16*** | |
| Focal isolate [means] | | | 1 | | 486.72*** | |
| Competitor isolate [*means*] | | | 1 | | 455.56*** | |
|  | |  | |  | |  |
| ^1^df indicates degrees of freedom, denominator df for *F* test is estimated using the between-within approximation. Df for likelihood ratio (LR) tests are equal to the difference in the number of parameters in the full and reduced models. | | | | | | |
| ^2^ Fixed effects are tested with approximate *F* tests. Random effects are tested using likelihood ratio LR tests; the LR test statistic is −2× (maximum likelihood of the test’s full model − maximum likelihood of the restricted model, from which the variance component being tested has been removed), and is distributed approximately chi-squared. In the tests of variance effects, variances are constrained to be equal in the reduced model. ^NS^P>0.1; ^∗∗∗^P<0.001. | | | | | | |

**Table S4. General linear mixed models assessing dependence of outcome of pairwise cross-streak competition among *P. aeruginosa* on inhibition phenotype.** Inhibition phenotype coded as whether or not the focal isolate inhibits the competitor, whether the competitor isolate inhibits the focal isolate, and the interaction between these two are fixed factors. Focal and competitor isolate were included as random factors. Inhibition was determined based on outcomes of pairwise inhibition assays (figure 2). Separate models are presented for experiments conducted at 25 °C and 37 °C.

| **Source** | **df^1^** | **Test statistic^2^** |
| --- | --- | --- |
| 25 °C | | |
| Focal isolate source | 1, 27 | 0.16^NS^ |
| Competitor source | 1, 27 | 1.50^NS^ |
| Focal isolate source x Competitor source | 1, 1716 | 3.72^NS^ |
| Focal isolate (Focal isolate source) [means] | 1 | 506.21*** |
| Focal isolate (Focal isolate source) [variances] | 1 | 19.13*** |
| Competitor isolate (Competitor source) [*means*] | 1 | 202.13*** |
| Competitor isolate (Competitor source) [*variances*] | 1 | 9.08 *** |
| 37 °C | | |
| Focal isolate source | 1, 27 | 0.61^NS^ |
| Competitor isolate source | 1, 27 | 1.40^NS^ |
| Focal isolate source x Competitor source | 1, 2143 | 1.66^NS^ |
| Focal isolate (Focal isolate source) [means] | 1 | 490.32*** |
| Focal isolate (Focal isolate source) [variances] | 1 | 8.15** |
| Competitor isolate (Competitor source) [*means*] | 1 | 472.01*** |
| Competitor isolate (Competitor source) [*variances*] | 1 | 1.30^NS^ |
|  |  |  |

| ^1^df indicates degrees of freedom, denominator df for *F* test is estimated using the between-within approximation. Df for likelihood ratio (LR) tests are equal to the difference in the number of parameters in the full and reduced models. |
| --- |
| ^2^Fixed effects are tested with approximate *F* tests. Random effects are tested using likelihood ratio LR tests; the LR test statistic is −2×(maximum likelihood of the test’s full model − maximum likelihood of the restricted model, from which the variance component being tested has been removed), and is distributed approximately chi-squared. In the tests of variance effects, variances are constrained to be equal in the reduced model. ^NS^P>0.1; ^∗∗^0.001<P<0.01; ^∗∗∗^P<0.001. |

**Table S5. General linear mixed models assessing dependence of outcome of pairwise cross-streak competition among *P. aeruginosa* on isolation source.** Sources (environment or CF) and their interactions are included as fixed factors and focal isolate and competitor isolate as random factors. Separate models are presented for experiments conducted at 25 °C and 37 °C.

| **Isolate Category** | **Isolate ID** | **S1_kill** | **S2_kill** | **S1.S2_imm** | **S3_kill** | **S3_imm** | **S4_kill** | **S4_imm** | **S5_kill** | **S6_kill** | **S6_imm** | **AP41_kill** | **AP41_imm** | **R-type** | **F-type** | **Total # genes** | **# of cytotoxic genes** | **# immunity genes** |
| --- | --- | --- | --- | --- | --- | --- | --- | --- | --- | --- | --- | --- | --- | --- | --- | --- | --- | --- |
| CF-associated | 1266 | 0 | 1 | 1 | 0 | 0 | 1 | 1 | 1 | 0 | 0 | 0 | 0 | 1 | 1 | 7 | 5 | 2 |
| CF-associated | 1428 | 0 | 1 | 1 | 0 | 0 | 1 | 1 | 1 | 0 | 0 | 0 | 0 | 1 | 1 | 7 | 5 | 2 |
| CF-associated | 3233 | 0 | 1 | 1 | 0 | 0 | 1 | 1 | 0 | 0 | 1 | 1 | 1 | 1 | 0 | 8 | 4 | 4 |
| CF-associated | 16-004 | 0 | 1 | 1 | 0 | 0 | 0 | 0 | 1 | 0 | 0 | 0 | 0 | 1 | 1 | 5 | 4 | 1 |
| CF-associated | 16-006 | 0 | 1 | 1 | 0 | 0 | 1 | 1 | 1 | 0 | 0 | 0 | 0 | 1 | 1 | 7 | 5 | 2 |
| CF-associated | 16-098 | 0 | 1 | 1 | 0 | 0 | 0 | 0 | 0 | 0 | 1 | 0 | 0 | 1 | 0 | 4 | 2 | 2 |
| CF-associated | 16-099 | 0 | 1 | 1 | 0 | 0 | 0 | 0 | 1 | 0 | 1 | 0 | 0 | 1 | 0 | 5 | 3 | 2 |
| CF-associated | 16-102 | 0 | 1 | 1 | 0 | 0 | 0 | 0 | 1 | 0 | 1 | 1 | 1 | 1 | 1 | 8 | 5 | 3 |
| CF-associated | 16-104 | 0 | 1 | 1 | 0 | 0 | 0 | 0 | 1 | 0 | 1 | 1 | 1 | 1 | 0 | 7 | 4 | 3 |
| CF-associated | 16-105 | 0 | 1 | 1 | 0 | 0 | 0 | 0 | 1 | 0 | 1 | 0 | 0 | 1 | 1 | 6 | 4 | 2 |
| CF-associated | 1055 | 1 | 0 | 1 | 0 | 0 | 0 | 0 | 0 | 0 | 1 | 0 | 0 | 1 | 1 | 5 | 3 | 2 |
| CF-associated | 1348 | 1 | 0 | 1 | 0 | 0 | 0 | 0 | 0 | 0 | 0 | 1 | 1 | 1 | 0 | 5 | 3 | 2 |
| CF-associated | 16-005 | 1 | 1 | 0 | 0 | 0 | 0 | 0 | 0 | 0 | 0 | 0 | 0 | 1 | 1 | 4 | 4 | 0 |
| CF-associated | 1814 | 0 | 1 | 0 | 0 | 0 | 0 | 1 | 0 | 0 | 1 | 1 | 1 | 1 | 0 | 6 | 3 | 3 |
| CF-associated | 2552 | 0 | 1 | 0 | 0 | 0 | 0 | 0 | 0 | 0 | 0 | 0 | 0 | 1 | 1 | 3 | 3 | 0 |
| CF-associated | 16-003 | 0 | 1 | 0 | 0 | 0 | 0 | 0 | 0 | 0 | 0 | 0 | 0 | 1 | 0 | 2 | 2 | 0 |
| CF-associated | 16-009 | 0 | 1 | 0 | 0 | 0 | 0 | 0 | 0 | 0 | 0 | 0 | 0 | 1 | 0 | 2 | 2 | 0 |
| CF-associated | 16-096 | 0 | 1 | 0 | 0 | 0 | 0 | 0 | 0 | 0 | 1 | 0 | 0 | 0 | 1 | 3 | 2 | 1 |
| CF-associated | 16-101 | 0 | 1 | 0 | 0 | 0 | 0 | 0 | 0 | 0 | 0 | 0 | 0 | 0 | 1 | 2 | 2 | 0 |
| CF-associated | 1790 | 1 | 0 | 0 | 0 | 0 | 0 | 0 | 0 | 0 | 0 | 0 | 0 | 1 | 1 | 3 | 3 | 0 |
| CF-associated | 1794 | 1 | 0 | 0 | 0 | 0 | 0 | 0 | 0 | 0 | 0 | 0 | 0 | 1 | 1 | 3 | 3 | 0 |
| CF-associated | 2092 | 1 | 0 | 0 | 0 | 0 | 0 | 0 | 0 | 0 | 0 | 0 | 0 | 1 | 1 | 3 | 3 | 0 |
| CF-associated | 16-001 | 1 | 0 | 0 | 0 | 0 | 1 | 0 | 1 | 0 | 1 | 0 | 0 | 1 | 1 | 6 | 5 | 1 |
| CF-associated | 16-002 | 1 | 0 | 0 | 0 | 0 | 0 | 0 | 0 | 0 | 1 | 0 | 0 | 0 | 1 | 3 | 2 | 1 |
| CF-associated | 16-007 | 1 | 0 | 0 | 0 | 0 | 0 | 0 | 0 | 0 | 1 | 0 | 0 | 1 | 0 | 3 | 2 | 1 |
| CF-associated | 16-103 | 1 | 0 | 0 | 0 | 0 | 0 | 0 | 0 | 0 | 1 | 0 | 0 | 1 | 1 | 4 | 3 | 1 |
| CF-associated | 3110 | 0 | 0 | 0 | 0 | 0 | 0 | 0 | 0 | 0 | 0 | 1 | 1 | 0 | 1 | 3 | 2 | 1 |
| CF-associated | 16-008 | 0 | 0 | 0 | 0 | 0 | 0 | 0 | 0 | 0 | 0 | 0 | 0 | 1 | 1 | 2 | 2 | 0 |
| CF-associated | 16-097 | 0 | 0 | 0 | 0 | 0 | 0 | 0 | 0 | 0 | 0 | 0 | 0 | 1 | 1 | 2 | 2 | 0 |
| CF-associated | 16-100 | 0 | 0 | 0 | 0 | 0 | 0 | 0 | 0 | 0 | 1 | 0 | 0 | 1 | 1 | 3 | 2 | 1 |
| Environmental | 1162 | 1 | 0 | 1 | 0 | 0 | 0 | 0 | 0 | 0 | 0 | 1 | 1 | 1 | 0 | 5 | 3 | 2 |
| Environmental | 1169 | 1 | 0 | 1 | 0 | 0 | 0 | 0 | 0 | 0 | 0 | 1 | 1 | 1 | 0 | 5 | 3 | 2 |
| Environmental | 1172 | 1 | 0 | 1 | 0 | 0 | 0 | 0 | 0 | 0 | 0 | 1 | 1 | 1 | 0 | 5 | 3 | 2 |
| Environmental | 1173 | 1 | 0 | 1 | 0 | 0 | 0 | 0 | 0 | 0 | 0 | 1 | 1 | 1 | 0 | 5 | 3 | 2 |
| Environmental | 1177 | 1 | 0 | 1 | 0 | 0 | 0 | 0 | 0 | 0 | 0 | 1 | 1 | 1 | 0 | 5 | 3 | 2 |
| Environmental | 1421 | 1 | 0 | 1 | 0 | 0 | 0 | 0 | 1 | 0 | 1 | 0 | 0 | 1 | 0 | 5 | 3 | 2 |
| Environmental | 1507 | 1 | 0 | 1 | 0 | 0 | 0 | 0 | 0 | 0 | 1 | 1 | 1 | 0 | 1 | 6 | 3 | 3 |
| Environmental | 1536 | 1 | 0 | 1 | 0 | 0 | 0 | 0 | 0 | 0 | 0 | 1 | 1 | 1 | 0 | 5 | 3 | 2 |
| Environmental | 1582 | 1 | 0 | 1 | 0 | 0 | 0 | 0 | 1 | 0 | 1 | 1 | 1 | 1 | 0 | 7 | 4 | 3 |
| Environmental | 1831 | 1 | 0 | 1 | 0 | 0 | 0 | 0 | 1 | 0 | 1 | 1 | 1 | 1 | 0 | 7 | 4 | 3 |
| Environmental | 1845 | 1 | 0 | 1 | 0 | 0 | 0 | 0 | 0 | 0 | 0 | 1 | 1 | 1 | 0 | 5 | 3 | 2 |
| Environmental | 2361 | 1 | 0 | 1 | 0 | 0 | 0 | 0 | 1 | 0 | 1 | 0 | 0 | 1 | 1 | 6 | 4 | 2 |
| Environmental | 2415 | 1 | 0 | 1 | 0 | 0 | 0 | 0 | 0 | 0 | 0 | 1 | 1 | 1 | 0 | 5 | 3 | 2 |
| Environmental | 2544 | 1 | 0 | 1 | 0 | 0 | 0 | 0 | 0 | 0 | 0 | 1 | 1 | 1 | 0 | 5 | 3 | 2 |
| Environmental | 2578 | 1 | 0 | 1 | 0 | 0 | 0 | 0 | 0 | 0 | 0 | 1 | 1 | 1 | 0 | 5 | 3 | 2 |
| Environmental | 2594 | 1 | 0 | 1 | 0 | 0 | 0 | 0 | 0 | 0 | 0 | 1 | 1 | 1 | 0 | 5 | 3 | 2 |
| Environmental | 2604 | 1 | 0 | 1 | 0 | 0 | 0 | 0 | 0 | 0 | 1 | 1 | 1 | 0 | 1 | 6 | 3 | 3 |
| Environmental | 2605 | 1 | 0 | 1 | 0 | 0 | 0 | 0 | 0 | 0 | 0 | 1 | 1 | 1 | 0 | 5 | 3 | 2 |
| Environmental | 2629 | 1 | 0 | 1 | 0 | 0 | 0 | 0 | 0 | 0 | 0 | 1 | 1 | 1 | 0 | 5 | 3 | 2 |
| Environmental | 2633 | 1 | 0 | 1 | 0 | 0 | 0 | 0 | 0 | 0 | 0 | 1 | 1 | 1 | 0 | 5 | 3 | 2 |
| Environmental | 2785 | 1 | 0 | 1 | 0 | 0 | 0 | 0 | 0 | 0 | 0 | 1 | 1 | 1 | 0 | 5 | 3 | 2 |
| Environmental | 3061 | 1 | 0 | 1 | 0 | 0 | 0 | 0 | 0 | 0 | 0 | 1 | 1 | 1 | 0 | 5 | 3 | 2 |
| Environmental | 3100 | 1 | 0 | 1 | 0 | 0 | 0 | 0 | 0 | 0 | 0 | 1 | 1 | 1 | 0 | 5 | 3 | 2 |
| Environmental | 16-012 | 1 | 0 | 1 | 0 | 0 | 0 | 0 | 0 | 0 | 0 | 1 | 1 | 1 | 0 | 5 | 3 | 2 |
| Environmental | 16-046 | 1 | 0 | 1 | 0 | 0 | 0 | 0 | 0 | 0 | 0 | 1 | 1 | 1 | 0 | 5 | 3 | 2 |
| Environmental | 16-065 | 1 | 1 | 0 | 0 | 0 | 0 | 0 | 0 | 0 | 0 | 0 | 0 | 0 | 1 | 3 | 3 | 0 |
| Environmental | 1027 | 0 | 1 | 0 | 0 | 0 | 0 | 0 | 0 | 0 | 0 | 0 | 0 | 1 | 1 | 3 | 3 | 0 |
| Environmental | 1062 | 0 | 1 | 0 | 0 | 0 | 0 | 0 | 0 | 0 | 0 | 0 | 0 | 1 | 0 | 2 | 2 | 0 |
| Environmental | 1068 | 0 | 1 | 0 | 0 | 0 | 0 | 0 | 0 | 0 | 0 | 0 | 0 | 0 | 1 | 2 | 2 | 0 |
| Environmental | 1078 | 0 | 1 | 0 | 0 | 0 | 0 | 0 | 0 | 0 | 0 | 0 | 0 | 0 | 1 | 2 | 2 | 0 |
| Environmental | 1081 | 0 | 1 | 0 | 0 | 0 | 0 | 0 | 0 | 0 | 0 | 0 | 0 | 0 | 1 | 2 | 2 | 0 |
| Environmental | 1091 | 0 | 1 | 0 | 0 | 0 | 0 | 0 | 0 | 0 | 0 | 0 | 0 | 0 | 1 | 2 | 2 | 0 |
| Environmental | 1106 | 0 | 1 | 0 | 0 | 0 | 0 | 1 | 0 | 0 | 0 | 0 | 0 | 0 | 1 | 3 | 2 | 1 |
| Environmental | 1150 | 0 | 1 | 0 | 0 | 0 | 1 | 0 | 0 | 0 | 0 | 0 | 0 | 1 | 0 | 3 | 3 | 0 |
| Environmental | 1274 | 0 | 1 | 0 | 0 | 0 | 0 | 0 | 0 | 0 | 0 | 0 | 0 | 1 | 0 | 2 | 2 | 0 |
| Environmental | 1276 | 0 | 1 | 0 | 0 | 0 | 0 | 0 | 0 | 0 | 0 | 0 | 0 | 0 | 1 | 2 | 2 | 0 |
| Environmental | 1435 | 0 | 1 | 0 | 0 | 0 | 0 | 0 | 0 | 0 | 0 | 0 | 0 | 1 | 0 | 2 | 2 | 0 |
| Environmental | 1439 | 0 | 1 | 0 | 0 | 0 | 0 | 0 | 0 | 0 | 0 | 0 | 0 | 0 | 1 | 2 | 2 | 0 |
| Environmental | 1505 | 0 | 1 | 0 | 0 | 0 | 0 | 0 | 0 | 0 | 0 | 1 | 1 | 1 | 1 | 5 | 4 | 1 |
| Environmental | 1537 | 0 | 1 | 0 | 0 | 0 | 0 | 0 | 0 | 0 | 0 | 0 | 0 | 1 | 0 | 2 | 2 | 0 |
| Environmental | 1538 | 0 | 1 | 0 | 0 | 0 | 0 | 0 | 0 | 0 | 0 | 0 | 0 | 1 | 0 | 2 | 2 | 0 |
| Environmental | 1607 | 0 | 1 | 0 | 0 | 0 | 0 | 0 | 0 | 0 | 0 | 0 | 0 | 0 | 1 | 2 | 2 | 0 |
| Environmental | 1609 | 0 | 1 | 0 | 0 | 0 | 0 | 0 | 0 | 0 | 0 | 0 | 0 | 1 | 0 | 2 | 2 | 0 |
| Environmental | 1618 | 0 | 1 | 0 | 0 | 0 | 0 | 0 | 0 | 0 | 0 | 0 | 0 | 1 | 0 | 2 | 2 | 0 |
| Environmental | 2151 | 0 | 1 | 0 | 0 | 0 | 0 | 0 | 0 | 0 | 0 | 0 | 0 | 0 | 1 | 2 | 2 | 0 |
| Environmental | 2253 | 0 | 1 | 0 | 0 | 0 | 0 | 0 | 0 | 0 | 0 | 0 | 0 | 0 | 1 | 2 | 2 | 0 |
| Environmental | 2299 | 0 | 1 | 0 | 0 | 0 | 0 | 0 | 0 | 0 | 0 | 0 | 0 | 0 | 1 | 2 | 2 | 0 |
| Environmental | 2500 | 0 | 1 | 0 | 0 | 0 | 0 | 0 | 0 | 0 | 0 | 0 | 0 | 0 | 1 | 2 | 2 | 0 |
| Environmental | 2521 | 0 | 1 | 0 | 0 | 0 | 0 | 0 | 0 | 0 | 0 | 0 | 0 | 0 | 1 | 2 | 2 | 0 |
| Environmental | 2588 | 0 | 1 | 0 | 0 | 0 | 0 | 0 | 0 | 0 | 1 | 1 | 1 | 1 | 1 | 6 | 4 | 2 |
| Environmental | 2590 | 0 | 1 | 0 | 0 | 0 | 0 | 0 | 0 | 0 | 1 | 1 | 1 | 1 | 1 | 6 | 4 | 2 |
| Environmental | 2597 | 0 | 1 | 0 | 0 | 0 | 0 | 0 | 0 | 0 | 0 | 0 | 0 | 1 | 1 | 3 | 3 | 0 |
| Environmental | 2620 | 0 | 1 | 0 | 0 | 0 | 0 | 0 | 0 | 0 | 0 | 0 | 0 | 0 | 1 | 2 | 2 | 0 |
| Environmental | 2674 | 0 | 1 | 0 | 0 | 0 | 0 | 0 | 0 | 0 | 0 | 0 | 0 | 0 | 1 | 2 | 2 | 0 |
| Environmental | 2684 | 0 | 1 | 0 | 0 | 0 | 0 | 0 | 0 | 0 | 1 | 1 | 1 | 1 | 1 | 6 | 4 | 2 |
| Environmental | 2836 | 0 | 1 | 0 | 0 | 0 | 0 | 0 | 0 | 0 | 0 | 0 | 0 | 0 | 1 | 2 | 2 | 0 |
| Environmental | 2877 | 0 | 1 | 0 | 0 | 0 | 0 | 0 | 0 | 0 | 0 | 0 | 0 | 0 | 1 | 2 | 2 | 0 |
| Environmental | 3091 | 0 | 1 | 0 | 0 | 0 | 0 | 0 | 0 | 0 | 1 | 1 | 1 | 1 | 1 | 6 | 4 | 2 |
| Environmental | 3173 | 0 | 1 | 0 | 0 | 0 | 0 | 0 | 0 | 0 | 0 | 0 | 0 | 0 | 1 | 2 | 2 | 0 |
| Environmental | 3209 | 0 | 1 | 0 | 0 | 0 | 0 | 0 | 0 | 0 | 0 | 0 | 0 | 0 | 1 | 2 | 2 | 0 |
| Environmental | 16-013 | 0 | 1 | 0 | 0 | 0 | 0 | 0 | 0 | 0 | 0 | 0 | 0 | 0 | 1 | 2 | 2 | 0 |
| Environmental | 16-054 | 0 | 1 | 0 | 0 | 0 | 0 | 0 | 0 | 0 | 0 | 0 | 0 | 0 | 1 | 2 | 2 | 0 |
| Environmental | 16-055 | 0 | 1 | 0 | 0 | 0 | 0 | 0 | 0 | 0 | 0 | 0 | 0 | 0 | 1 | 2 | 2 | 0 |
| Environmental | 16-064 | 0 | 1 | 0 | 0 | 0 | 0 | 0 | 0 | 0 | 0 | 1 | 0 | 0 | 1 | 3 | 3 | 0 |
| Environmental | 2670 | 1 | 0 | 0 | 0 | 0 | 0 | 0 | 0 | 0 | 0 | 0 | 0 | 1 | 1 | 3 | 3 | 0 |
| Environmental | 2967 | 1 | 0 | 0 | 0 | 0 | 0 | 0 | 0 | 0 | 1 | 0 | 0 | 1 | 0 | 3 | 2 | 1 |
| Environmental | 3199 | 1 | 0 | 0 | 0 | 0 | 0 | 0 | 0 | 0 | 1 | 0 | 0 | 1 | 0 | 3 | 2 | 1 |
| Environmental | 16-019 | 1 | 0 | 0 | 0 | 0 | 0 | 0 | 0 | 0 | 1 | 0 | 0 | 1 | 0 | 3 | 2 | 1 |
| Environmental | 16-020 | 1 | 0 | 0 | 0 | 0 | 0 | 0 | 0 | 0 | 1 | 0 | 0 | 1 | 0 | 3 | 2 | 1 |
| Environmental | 1005 | 0 | 0 | 0 | 0 | 0 | 0 | 0 | 0 | 0 | 0 | 1 | 1 | 0 | 1 | 3 | 2 | 1 |
| Environmental | 1258 | 0 | 0 | 0 | 0 | 0 | 0 | 0 | 0 | 0 | 0 | 0 | 0 | 0 | 1 | 1 | 1 | 0 |
| Environmental | 1904 | 0 | 0 | 0 | 0 | 0 | 0 | 0 | 0 | 0 | 0 | 1 | 1 | 0 | 1 | 3 | 2 | 1 |
| Environmental | 1943 | 0 | 0 | 0 | 0 | 0 | 0 | 0 | 0 | 0 | 0 | 1 | 1 | 0 | 1 | 3 | 2 | 1 |
| Environmental | 1952 | 0 | 0 | 0 | 0 | 0 | 0 | 0 | 0 | 0 | 0 | 0 | 0 | 0 | 0 | 0 | 0 | 0 |
| Environmental | 2004 | 0 | 0 | 0 | 0 | 0 | 0 | 0 | 0 | 0 | 0 | 0 | 0 | 1 | 1 | 2 | 2 | 0 |
| Environmental | 2048 | 0 | 0 | 0 | 1 | 0 | 0 | 0 | 0 | 0 | 0 | 0 | 0 | 1 | 0 | 2 | 2 | 0 |
| Environmental | 2230 | 0 | 0 | 0 | 1 | 0 | 0 | 0 | 0 | 0 | 0 | 0 | 0 | 1 | 0 | 2 | 2 | 0 |
| Environmental | 2304 | 0 | 0 | 0 | 0 | 0 | 0 | 0 | 0 | 0 | 0 | 0 | 0 | 0 | 1 | 1 | 1 | 0 |
| Environmental | 2562 | 0 | 0 | 0 | 1 | 0 | 0 | 0 | 0 | 0 | 0 | 0 | 0 | 1 | 1 | 3 | 3 | 0 |
| Environmental | 2563 | 0 | 0 | 0 | 1 | 0 | 0 | 0 | 0 | 0 | 0 | 0 | 0 | 1 | 1 | 3 | 3 | 0 |
| Environmental | 2630 | 0 | 0 | 0 | 1 | 1 | 1 | 1 | 1 | 0 | 0 | 0 | 0 | 1 | 1 | 7 | 5 | 2 |
| Environmental | 2696 | 0 | 0 | 0 | 1 | 0 | 0 | 1 | 0 | 0 | 0 | 0 | 0 | 1 | 1 | 4 | 3 | 1 |
| Environmental | 2704 | 0 | 0 | 0 | 1 | 0 | 0 | 0 | 0 | 0 | 0 | 0 | 0 | 1 | 0 | 2 | 2 | 0 |
| Environmental | 3151 | 0 | 0 | 0 | 1 | 1 | 1 | 1 | 1 | 0 | 0 | 0 | 0 | 1 | 1 | 7 | 5 | 2 |
| Environmental | 3232 | 0 | 0 | 0 | 0 | 0 | 0 | 0 | 0 | 0 | 0 | 1 | 1 | 0 | 1 | 3 | 2 | 1 |
| Environmental | 16-023 | 0 | 0 | 0 | 0 | 0 | 0 | 0 | 0 | 0 | 0 | 1 | 0 | 1 | 0 | 2 | 2 | 0 |

**Table S6. Pyocin haplotype of the 116 *P. aeruginosa* isolates included in this study.**
